# Supplementary material for: Interventional Influence of the Intestinal Microbiome Through Dietary Intervention and Bowel Cleansing Might Improve Motor Symptoms in Parkinson’s Disease
Source: Cells. 2020 Feb 6;9(2):376. doi: 10.3390/cells9020376 (PMC7072275; doi:10.3390/cells9020376)
Supplement: Supplementary file 1 [file cells-09-00376-s001.pdf]

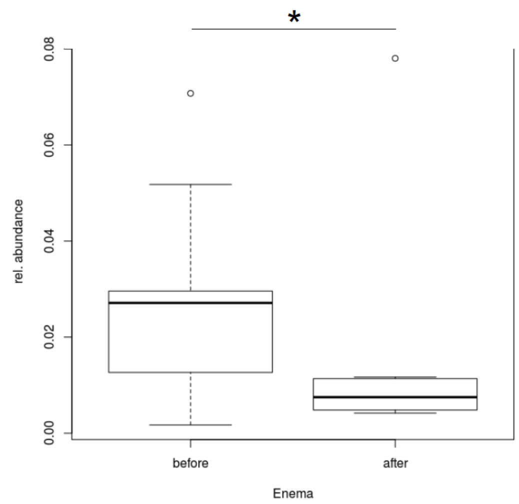


**Figure S1.** Relative abundance of Clostridiaceae before and after therapy (*= p<0.05; **= p<0.01).

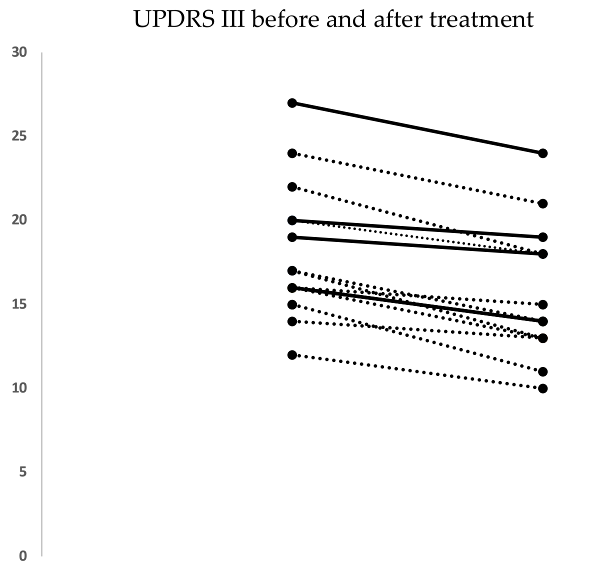


**Figure S2.** UPDRS III before and after the interventional interval of 14 days. The dotted line represents the intervention group with enema and vegetarian diet, while the solid line represents the group with vegetarian diet alone.

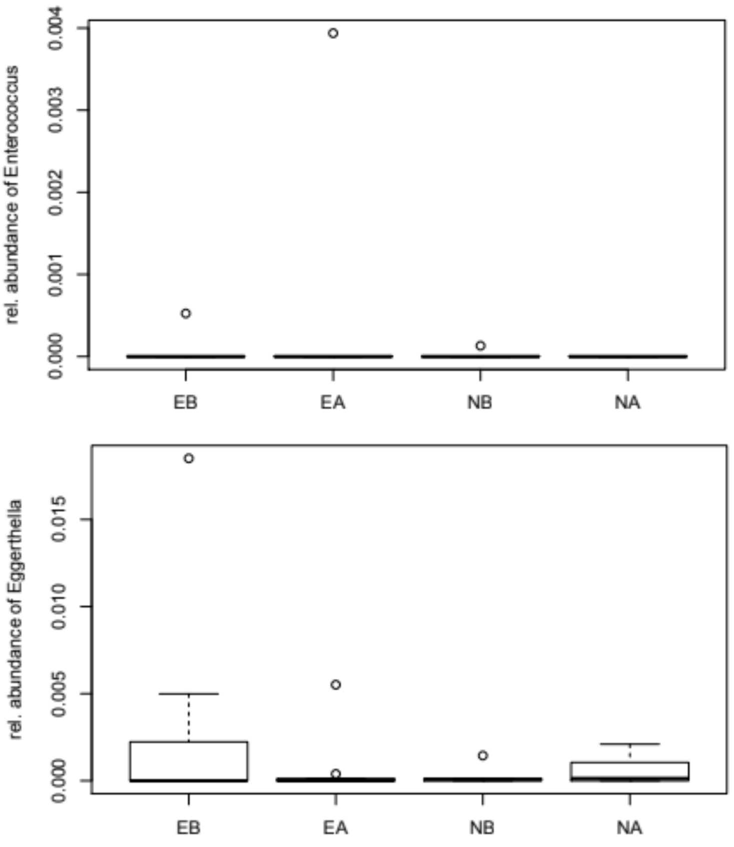


**Figure S3.** Relative abundance of the level in patients with Parkinson disease before and after intervention. EB: enema before, EA: enema after intervention; NB: nutrition before, NA: nutrition after intervention.

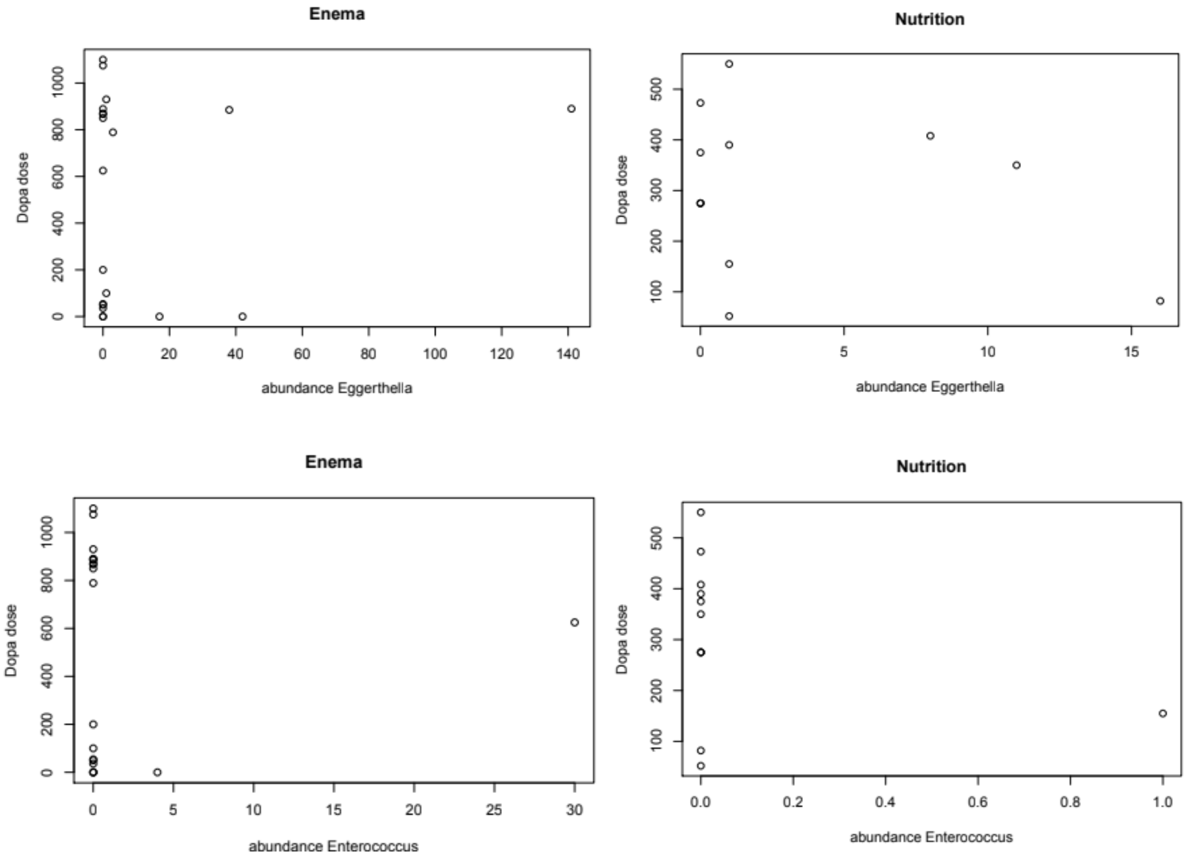


**Figure S4.** Correlation between the rel. abundance of *Eggerthela lenta* and the cum. levodopa dose [mg] at the top for both groups. Correlation between the rel. abundance of *Enterococcus faecalis* and the cum. levodopa dose [mg] at the bottom for both groups.
